# Telehealth Availability and Use of Related Technologies Among Medicare-Enrolled Cancer Survivors: Cross-sectional Findings From the Onset of the COVID-19 Pandemic

**DOI:** 10.2196/34616

**Published:** 2022-01-25

**Authors:** Yuki Lama, Amy J Davidoff, Robin C Vanderpool, Roxanne E Jensen

**Affiliations:** 1 Health Communication and Informatics Research Branch Behavioral Research Program National Cancer Institute Bethesda, MD United States; 2 Healthcare Assessment Research Branch Healthcare Delivery Research Program National Cancer Institute Bethesda, MD United States; 3 Outcomes Research Branch Healthcare Delivery Research Program National Cancer Institute Bethesda, MD United States

**Keywords:** cancer survivor, Medicare, telehealth, COVID-19, availability, use, elderly, older adults, cancer, sociodemographic, internet, communication, population, access

## Abstract

**Background:**

There has been rapid integration of telehealth into care delivery during the COVID-19 pandemic. However, little is known about technology ownership, internet access and use for communication, and telehealth availability among cancer survivors, particularly those enrolled in Medicare.

**Objective:**

This study aims to identify sociodemographic associations with technology ownership, internet access and use for communication, and telehealth availability in a population-based sample of Medicare-enrolled cancer survivors.

**Methods:**

Data are from the Medicare Current Beneficiary Survey COVID-19 Summer 2020 Supplement administered between June 10 and July 15, 2020. Analyses were restricted to beneficiaries who reported a prior (nonskin) cancer diagnosis and a usual source of care (N=2044). Dichotomous outcomes included technology ownership, internet access, internet use for communication, and telehealth availability from providers. Sociodemographic correlates included sex, age, race/ethnicity, Medicare/Medicaid dual enrollment, rurality, census region, and self-reported comorbidities.

**Results:**

Over half (957/2044, 53%) of cancer survivors reported using the internet for communication purposes, and 62% (1218/2044) reported that their usual provider had telehealth services available. Using the internet for communication purposes was reported less frequently for rural compared to urban survivors (adjusted probability of 28% vs 46%; *P*<.001) and for Hispanic and Black survivors compared to non-Hispanic White survivors (29%, 31%, and 44%, respectively; all *P*<.01). Rural survivors reported lower telehealth availability (53% vs 63%; *P*<.001); no significant differences in telehealth availability were identified by race/ethnicity.

**Conclusions:**

During the COVID-19 pandemic, study findings highlight a complex digital divide among Medicare beneficiaries with a history of cancer related to device ownership necessary for telehealth, internet access and use for communication, and reports of providers having telehealth available. Multilevel approaches are needed to increase equitable telehealth availability and use for cancer survivors. Suggested strategies include increasing broadband internet access to providers and patients in at-risk communities, supporting telehealth implementation among providers that serve populations with known health disparities, raising awareness of providers’ available telehealth services among patients, and screening for technology use and provision of telehealth-related technical assistance among older and historically underserved cancer survivors.

## Introduction

Throughout the COVID-19 pandemic, there has been a substantial increase in telehealth, defined as “the exchange of medical information from one site to another through electronic communication to improve a patient’s health,” to deliver health care services [1-3]. Increased telehealth use was motivated by the need to limit COVID-19 exposure, particularly among vulnerable patient populations, and facilitated by increased familiarity with teleconferencing and changes in reimbursement and regulatory policies [2]. Cancer survivors are likely to be particularly vulnerable to access to care obstacles associated with COVID-19: they tend to be older and may have comorbidities or immunosuppression that heightens both risk and consequences of infection [4]. As a result, telehealth can provide particular benefit by reducing logistical barriers to timely cancer-related care [5]. Prior research has identified barriers to successful telehealth use for adults generally, including lack of computer and internet access, and limited digital literacy skills needed to negotiate video log-on processes [6]. Previous research demonstrates that characteristics such as older age, rurality, and lower income were associated with less access to and engagement with telehealth prepandemic [7-9]. However, assessing whether these patterns apply to cancer survivors, and persisted during the onset of the pandemic, remains underexplored. Therefore, this exploratory study’s aim was to identify sociodemographic correlates of technology ownership, internet access, internet use for communication, and telehealth availability, captured early during the COVID-19 pandemic among a nationally representative sample of Medicare beneficiaries with a cancer history in the United States.

## Methods

### Data Source and Sample

The Medicare Current Beneficiary Survey COVID-19 Summer 2020 Supplement, sponsored by the Centers for Medicare and Medicaid Services, is a telephone survey of community-dwelling Medicare beneficiaries administered between June 10 to July 15, 2020. Individuals who are 65 years or older, disabled, or have end-stage renal disease (ESRD) are eligible for Medicare [10]. Survey weights represent the population of beneficiaries continuously enrolled in Medicare from January through summer 2020. Selected beneficiaries reported prior non–skin cancer diagnosis and a usual source of care other than urgent care or emergency departments.

### Outcome Variables

We evaluated four dichotomous measures: (1) technology ownership (“Do you own or use any of the following: desktop or laptop, smartphone, or tablet?”), (2) internet access (“Do you have access to the internet?”), (3) internet use for communication (“Have you ever participated in video or voice calls or conferencing over the internet, such as with Skype or FaceTime?”), and (4) telehealth availability (“Does your usual provider offer telephone or video appointments, so that you don’t need to physically visit their office or facility?”).

### Independent Variables

We assessed the role of sociodemographic characteristics, including age, race/ethnicity, Medicare/Medicaid dual enrollment (an indicator of poverty) [11], and rurality defined by metropolitan statistical area, adjusting for sex, census region, and self-reported comorbidities.

### Statistical Analyses

Descriptive statistics and bivariate comparisons were generated for each outcome. Multivariable logistic regression models estimated the effects of the independent variables on each outcome. Results related to age, race/ethnicity, Medicare/Medicaid dual enrollment, and rurality are reported as adjusted predicted (marginal) probabilities. All estimates were weighted, and analyses used SAS 9.4 (SAS Institute, Inc) procedures to adjust for complex survey design (PROC SURVEYFREQ, SURVEYMEANS, and SURVEYLOGISTIC). Statistical tests were 2-sided with α=.05. As Medicare Current Beneficiary Survey data are publicly available and deidentified, the study was not considered to be human participant research.

## Results

The sample of Medicare-enrolled cancer survivors was 57% (1144/2044) female, 41% (1192/2044) 75 years or older, and 79% (1638/2044) non-Hispanic White (n=2044; weighted n=9,941,910). Over half (957/2044, 53%) used the internet for communication and 62% (1218/2044) reported telehealth availability (Table 1). Please note that the percentages reported are weighted, while the sample sizes are unweighted, and hence, percentages may differ.

**Table 1:** Sample characteristics of Medicare beneficiaries reporting a cancer history and a usual source of care as assessed by the Medicare Current Beneficiary Survey (N=2044).

Characteristic			Unweighted n		Weighted %
**Sex**					
	Male	900		43.1	
	Female	1144		56.9	
**Age group (years)**					
	<65	201		10.8	
	65-74	651		48.1	
	≥75	1192		41.1	
**Race/ethnicity**					
	White non-Hispanic	1638		79.1	
	Black non-Hispanic	135		7.7	
	Hispanic	159		6.1	
	Other/unknown	112		7.1	
**Dual Medicare/Medicaid enrollment (2019)**					
	Nondual Medicare/Medicaid enrollment	1747		87.7	
	Any dual Medicare/Medicaid enrollment	297		12.3	
**Metropolitan statistical area residence^a^**					
	Urban	1542		79.6	
	Rural	502		20.4	
**Census region**					
	Northeast	372		18.2	
	Midwest	461		21.3	
	South	819		40.0	
	West	392		20.6	
**Comorbidities**					
	0 conditions	458		24.2	
	1 condition	653		32.3	
	≥2 conditions	933		43.5	
**Outcomes**					
	Technology ownership (computer, smartphone, tablet)	1588		83.3	
	Internet access	1604		82.9	
	Internet use for communication	957		53.0	
	Telehealth availability	1218		62.0	

^a^Metropolitan statistical area residence defined by the Office of Management and Budget as having at least one urbanized area with a minimum population of 50,000.

### Technology Ownership, Internet Access, and Internet Use for Communication

Older age, rural residence, dual Medicare/Medicaid enrollment, and non-Hispanic Black or Hispanic race/ethnicity were associated with lower probabilities of owning technology (Table 2), internet access (Table 3), and internet use for communication (all *P*<.05; Table 4). Compared to urban cancer survivors, rural survivors had lower predicted probabilities of technology ownership (67% vs 82%; *P*<.001; Table 2), internet access (58% vs 79%; *P*<.001; Table 3), and internet use for communication (28% vs 46%; *P*<.001; Table 4). Compared to non-Hispanic Whites, Hispanic and Black survivors had lower technology ownership (67% vs 82%, *P*<.001; 65% vs 82%, *P*=.005, respectively), internet access (56% vs 81%, *P*<.001; 52% vs 82%, *P*<.001, respectively), and internet use for communication (29% vs 44%, *P*<.001; 31% vs 44%, *P*=.002, respectively). Compared to nondual enrolled, dual Medicare/Medicaid enrolled beneficiaries had lower technology ownership (60% vs 86%; *P*<.001), internet access (53% vs 83%; *P*<.001), and internet use for communication (26% vs 48%; *P*<.001).

**Table 2:** Factors associated with technology ownership among Medicare Current Beneficiary Survey respondents with a history of cancer and a usual source of care (N=2044).

Characteristic			Own computer, tablet, or smartphone				
			Predicted probability		aOR^a^ (95% CI)		*P* value
**Metropolitan statistical area residence^b^**							
	Urban (reference)	0.82		N/A^c^		N/A	
	Rural	0.67		0.44 (0.31-0.62)		<.001	
**Age group (years)**							
	65-74 (reference)	0.79		N/A		N/A	
	<65	0.88		2.06 (1.07-3.94)		.03	
	≥75	0.50		0.27 (0.19-0.39)		<.001	
**Sex**							
	Male (reference)	0.72		N/A		N/A	
	Female	0.75		1.14 (0.89-1.47)		.32	
**Race/ethnicity**							
	White non-Hispanic (reference)	0.82		N/A		N/A	
	Black non-Hispanic	0.65		0.40 (0.21-0.75)		.005	
	Hispanic	0.67		0.44 (0.26-0.75)		<.001	
	Other/unknown	0.83		1.05 (0.51-2.13)		.90	
**Dual Medicare/Medicaid enrollment (2019)**							
	Nondual Medicare/Medicaid enrollment (reference)	0.86		N/A		N/A	
	Any enrollment Medicare/Medicaid	0.60		0.25 (0.15-0.41)		<.001	
**Census region**							
	Midwest (reference)	0.71		N/A		N/A	
	Northeast	0.72		1.05 (0.68-1.63)		.83	
	South	0.72		1.09 (0.74-1.60)		.66	
	West	0.84		2.23 (1.41-3.53)		<.001	
**Comorbidities**							
	0 (reference)	0.77		N/A		N/A	
	1	0.74		0.85 (0.56-1.29)		.45	
	≥2	0.74		0.85 (0.60-1.21)		.36	

^a^aOR: adjusted odds ratio.

^b^Metropolitan statistical area residence defined by the Office of Management and Budget as having at least one urbanized area with a minimum population of 50,000.

^c^N/A: not applicable.

**Table 3:** Factors associated with internet access among Medicare Current Beneficiary Survey respondents with a history of cancer and a usual source of care (N=2044).

Characteristic			Internet access			
			Predicted probability	aOR^a^ (95% CI)		*P* value
**Metropolitan statistical area residence^b^**						
	Metro (reference)	0.79		N/A^c^	N/A	
	Nonmetro	0.58		0.35 (0.25-0.50)	<.001	
**Age group (years)**						
	65-74 (reference)	0.74		N/A	N/A	
	<65	0.83		1.73 (0.84-3.55)	.14	
	≥75	0.46		0.30 (0.22-0.42)	<.001	
**Sex**						
	Male (reference)	0.70		N/A	N/A	
	Female	0.69		0.92 (0.73-1.17)	.49	
**Race/ethnicity**						
	White non-Hispanic (reference)	0.81		N/A	N/A	
	Black non-Hispanic	0.52		0.26 (0.13-0.52)	<.001	
	Hispanic	0.56		0.30 (0.18-0.51)	<.001	
	Other/unknown	0.82		1.04 (0.55-1.94)	.91	
**Dual Medicare/Medicaid enrollment (2019)**						
	Nondual Medicare/Medicaid enrollment (reference)	0.83		N/A	N/A	
	Any enrollment Medicare/Medicaid	0.53		0.24 (0.16-0.34)	<.001	
**Census region**						
	Midwest (reference)	0.65		N/A	N/A	
	Northeast	0.63		0.94 (0.60-1.45)	.76	
	South	0.67		1.11 (0.73-1.71)	.62	
	West	0.81		2.35 (1.29-4.28)	.01	
**Comorbidities**						
	0 (reference)	0.71		N/A	N/A	
	1	0.69		0.91 (0.61-1.34)	.62	
	≥2	0.68		0.88 (0.58-1.32)	.52	

^a^aOR: adjusted odds ratio.

^b^Metropolitan statistical area residence defined by the Office of Management and Budget as having at least one urbanized area with a minimum population of 50,000.

^c^N/A: not applicable.

**Table 4:** Factors associated with internet use for communication among Medicare Current Beneficiary Survey respondents with a history of cancer and a usual source of care (N=2044).

Characteristic		Internet use for communication		
		Predicted probability	aOR^a^ (95% CI)	*P* value
**Metropolitan statistical area residence^b^**				
	Metro (reference)	0.46	N/A^c^	N/A
	Nonmetro	N/A	0.45 (0.34-0.59)	<.001
**Age group (years)**				
	65-74 (reference)	0.42	N/A	N/A
	<65	0.47	1.22 (0.84-1.77)	.30
	≥75	0.22	0.38 (0.31-0.47)	<.001
**Sex**				
	Male (reference)	0.34	N/A	N/A
	Female	0.38	1.19 (0.96-1.47)	.11
**Race/ethnicity**				
	White non-Hispanic (reference)	0.44	N/A	N/A
	Black non-Hispanic	0.31	0.55 (0.33-0.92)	.002
	Hispanic	0.29	0.52 (0.34-0.79)	<.001
	Other/unknown	0.42	0.93 (0.58-1.50)	.76
**Dual Medicare/Medicaid enrollment (2019)**				
	Nondual Medicare/Medicaid enrollment (reference)	0.48	N/A	N/A
	Any enrollment Medicare/Medicaid	0.26	0.39 (0.26-0.59)	<.001
**Census region**				
	Midwest (reference)	0.36	N/A	N/A
	Northeast	0.34	0.93 (0.65-1.32)	.66
	South	0.34	0.94 (0.71-1.24)	.65
	West	0.42	1.33 (0.94-1.89)	.11
**Comorbidities**				
	0 (reference)	0.39	N/A	N/A
	1	0.37	0.90 (0.68-1.19)	.46
	≥2	0.34	0.79 (0.61-1.03)	.08

^a^aOR: adjusted odds ratio.

^b^Metropolitan statistical area residence defined by the Office of Management and Budget as having at least one urbanized area with a minimum population of 50,000.

^c^N/A: not applicable.

### Telehealth Availability

Older age, rural residence, and dual Medicare/Medicaid enrollment were associated with lower probabilities of telehealth availability (Table 5). Compared to urban survivors, rural survivors had lower predicted probability of telehealth availability (53% vs 63%; *P*<.001). Telehealth availability was not associated with race/ethnicity. Compared to nondual enrollees, dual Medicare/Medicaid-enrolled beneficiaries had lower probability of being offered telehealth (53% vs 63%; *P*=.009).

**Table 5:** Factors associated with telehealth availability among Medicare Current Beneficiary Survey respondents with a history of cancer and a usual source of care (N=2044).

Characteristic			Telehealth availability				
			Predicted probability		aOR^a^ (95% CI)		*P* value
**Metropolitan statistical area residence^b^**							
	Metro (reference)	0.63		N/A^c^		N/A	
	Nonmetro	0.53		0.68 (0.53-0.87)		<.001	
**Age group (years)**							
	65-74 (reference)	0.60		N/A		N/A	
	<65	0.64		1.18 (0.78-1.79)		.40	
	≥75	0.50		0.67 (0.54-0.83)		<.001	
**Sex**							
	Male (reference)	0.59		N/A		N/A	
	Female	0.58		0.94 (0.74-1.21)		.65	
**Race/ethnicity**							
	White non-Hispanic (reference)	0.59		N/A		N/A	
	Black non-Hispanic	0.56		0.89 (0.52-1.50)		.65	
	Hispanic	0.59		1.01 (0.67-1.52)		.95	
	Other/unknown	0.60		1.06 (0.63-1.77)		.83	
**Dual Medicare/Medicaid enrollment (2019)**							
	Nondual Medicare/Medicaid enrollment (reference)	0.63		N/A		N/A	
	Any enrollment Medicare/Medicaid	0.53		0.66 (0.48-0.90)		.009	
**Census region**							
	Midwest (reference)	0.56		N/A		N/A	
	Northeast	0.56		1.01 (0.74-1.38)		.96	
	South	0.51		0.86 (0.65-1.13)		.27	
	West	0.70		1.88 (1.27-2.79)		.002	
**Comorbidities**							
	0 (reference)	0.56		N/A		N/A	
	1	0.58		1.07 (0.83-1.39)		.60	
	≥2	0.61		1.26 (0.95-1.69)		.11	

^a^aOR: adjusted odds ratio.

^b^Metropolitan statistical area residence defined by the Office of Management and Budget as having at least one urbanized area with a minimum population of 50,000.

^c^N/A: not applicable.

## Discussion

### Principal Findings

Early during the COVID-19 pandemic, we found that over 80% of Medicare-enrolled cancer survivors owned the necessary technology for telehealth encounters, but only half had experience using the internet for communication; almost two-thirds of survivors reported that their usual provider offered telehealth. Consistent with previous research [12-14], study findings highlight a complex digital divide related to telehealth availability and technology ownership and use, particularly among older, Black, Hispanic, lower-income, and rural cancer survivors. Despite the potential of telehealth to meet the unique health care needs of cancer survivors (eg, surveillance, comorbidities, and primary and survivorship care), some patient groups face greater barriers to technology access. These patterned differences in use and access underscore a need to engage multilevel interventions to mitigate the underlying barriers to telehealth use. These results have implications for clinicians, patient advocates, and policy makers as they seek to improve access to care for vulnerable cancer survivors, particularly as the COVID-19 pandemic continues and telehealth becomes an increasingly important bridge between patients and providers.

Study findings highlight gaps in reported telehealth availability, raising concerns that some providers may have limited telehealth infrastructure [15]. Given the increasingly important role of telehealth to access services, clinicians may need to enhance their practices’ telehealth capabilities and clinical workflow by providing additional staff support during video log-on and follow-up processes. New procedures may be needed to assess and refer patients to community resources that can augment technology access and telehealth literacy [16]. Patient advocates and policy makers can support clinician efforts to engage patients via telehealth through continued reimbursement of telehealth visits that support technical and staff requirements. Legislation supporting reimbursement of telehealth services beyond the pandemic, including audio-only telehealth visits, is important in providing equitable access to care among older, rural cancer survivors living in poverty [17].

Gaps in patient access to technology need to be considered within the broader context of structural inequities and policies to address them. Data suggest limited broadband internet access is more prevalent among rural residents, adults 65 years and older, minority populations, and communities of lower socioeconomic status, the same populations that experience disparities in access to cancer survivorship care [18-20]. In parallel with supporting clinicians, ongoing efforts by policy makers to expand broadband access will be essential to reducing disparities in telehealth access. Findings suggest systemic factors may be influencing technology ownership, internet access and use for communication, and telehealth availability, necessitating further monitoring of telehealth in health care delivery to ensure that existing inequities in survivorship care are not exacerbated as telehealth availability increases.

### Limitations and Future Research

The cross-sectional data are from June and July 2020, and do not reflect the population’s telehealth experience over the course of the COVID-19 pandemic. Technology access and use and provider telehealth availability were self-reported and may be subject to bias; the survey did not measure telehealth use or difficulties with technology. The analytic sample included individuals aged <65 years, a special group of younger patients who are either disabled or have ESRD, and may not be representative of other adult cancer survivors aged <65 years. Further, although sample respondents reported a cancer history, we cannot ascertain if cancer was an active health problem. Despite these limitations, the study draws on a large, population-based sample of cancer survivors, with timely and targeted questions addressing telehealth use early during the COVID-19 pandemic. Future research is needed to assess cancer survivors’ experience with telehealth use as the pandemic continues. In addition, research should examine providers’ experience with offering their patients telehealth and challenges in serving historically vulnerable populations of cancer survivors. More research is needed to understand the telehealth needs and preferences of Medicare-enrolled cancer survivors, particularly those facing barriers to accessing and using technology necessary for telehealth.

### Conclusion

This study captures disparities in telehealth availability and related technological requirements during the COVID-19 pandemic among Medicare-enrolled cancer survivors. Developing and testing multilevel solutions for the “double-burden” of lack of technological access and disparities in access to health care are important to ensure existing inequities in survivorship care are not exacerbated as telehealth becomes more embedded into postpandemic health care delivery models.

## Abbreviations

ESRD: end-stage renal disease
